# Loss of matrilin 1 does not exacerbate the skeletal phenotype in a mouse model of multiple epiphyseal dysplasia caused by a *Matn3* V194D mutation

**DOI:** 10.1002/art.33486

**Published:** 2012-05

**Authors:** Peter A Bell, Katarzyna A Piróg, Maryline Fresquet, David J Thornton, Raymond P Boot-Handford, Michael D Briggs

**Affiliations:** University of ManchesterManchester, UK

## Abstract

**Objective:**

Mutations in matrilin 3 can result in multiple epiphyseal dysplasia (MED), a disease characterized by delayed and irregular bone growth and early-onset osteoarthritis. Although intracellular retention of the majority of mutant matrilin 3 was previously observed in a murine model of MED caused by a *Matn3* V194D mutation, some mutant protein was secreted into the extracellular matrix. Thus, it was proposed that secretion of mutant matrilin 3 may be dependent on the formation of hetero-oligomers with matrilin 1. The aim of this study was to investigate the hypothesis that deletion of matrilin 1 would abolish the formation of matrilin 1/matrilin 3 hetero-oligomers, eliminate the secretion of mutant matrilin 3, and influence disease severity.

**Methods:**

Mice with a *Matn3* V194D mutation were crossed with *Matn1*-null mice, generating mice that were homozygous for V194D and null for matrilin 1. This novel mouse was used for in-depth phenotyping, while cartilage and chondrocytes were studied both histochemically and biochemically.

**Results:**

Endochondral ossification was not disrupted any further in mice with a double V194D mutation compared with mice with a single mutation. A similar proportion of mutant matrilin 3 was present in the extracellular matrix, and the amount of retained mutant matrilin 3 was not noticeably increased. Retained mutant matrilin 3 formed disulfide-bonded aggregates and caused the co-retention of matrilin 1.

**Conclusion:**

We showed that secretion of matrilin 3 V194D mutant protein is not dependent on hetero-oligomerization with matrilin 1, and that the total ablation of matrilin 1 expression has no impact on disease severity in mice with MED. Mutant matrilin 3 oligomers form non-native disulfide-bonded aggregates through the misfolded A domain.

The chondrodysplasias are a group of >450 different phenotypes (1) that occur when endochondral bone growth is disrupted (2). The clinical presentation of chondrodysplasia varies from mild to lethal but is generally distinguished by disproportionate short stature. Multiple epiphyseal dysplasia (MED) is characterized by delayed and irregular endochondral ossification and early-onset osteoarthritis (3, 4). MED is genetically heterogeneous, and autosomal-dominant forms of the disease arise from mutations in the genes encoding matrilin 3, cartilage oligomeric matrix protein (COMP), and type IX collagen (3–5). Although present in other musculoskeletal tissues, matrilin 3, COMP, and type IX collagen are expressed primarily by chondrocytes and have been shown to interact with each other, both in vitro and in vivo (6–13).

The matrilins are a family of 4 noncollagenous extracellular matrix (ECM) proteins. Each matrilin monomer comprises a C-terminal α-helical coiled-coil oligomerization domain, a varying number of epidermal growth factor–like domains, and 1 (matrilin 3) or 2 (matrilins 1, 2, and 4) von Willebrand factor A domains (6, 14). Matrilin proteins bind to numerous ECM components, including type II collagen, type IX collagen, aggrecan, COMP, biglycan, and decorin, and are believed to act as adaptor proteins in the ECM (6).

More than 20 different missense mutations within the matrilin 3 gene have been shown to cause autosomal-dominant MED, and *MATN3* mutations are believed to account for ∼20% of all cases of autosomal-dominant MED (5). The majority of *MATN3* mutations affect residues that are located within the central β-sheet of the single A domain of matrilin 3 (5, 15–17), while a smaller proportion of mutations that affect residues in the α-helices of the same domain have recently been described (5, 18, 19).

Several different cell culture model systems have been used in the past to study the effect of matrilin 3 mutations in vitro (15, 19, 20). The most striking effect of *MATN3* mutations was the intracellular retention of mutant protein in cells in culture and also in patient cartilage. This distinctive finding was recently verified in a murine model of MED caused by a *Matn3* V194D mutation, which replicated the human phenotype by exhibiting mild short-limbed dwarfism (21, 22). In this murine model, mutant matrilin 3 was retained within the endoplasmic reticulum (ER) of chondrocytes from the growth plates of endochondral bones. The retention of mutant matrilin 3 elicited a conventional unfolded protein response characterized by the up-regulation of classic chaperone proteins such as BiP/glucose-regulated protein 78-kd, calnexin, and calreticulin, as well as less-characterized ER stress–associated genes such as *Creld2* and *Armet*/*Manf* (22).

Despite intracellular retention of the majority of mutant matrilin 3 in vivo, a small proportion of mutant protein was secreted into the ECM, even in mice that were homozygous for the V194D mutation (21). The structural and/or functional effect of this mutant matrilin 3 in the ECM is not known, nor is the extent to which its presence in the ECM might contribute to the chondrodysplasia phenotype. Given the widespread literature describing the hetero-oligomerization of matrilin 3 with matrilin 1 (23–26), we considered the possibility that the secretion of mutant matrilin 3 may be dependent on its oligomerization into heterotetramers containing wild-type matrilin 1.

We hypothesized that deletion of matrilin 1, which would abolish the formation of matrilin 1/matrilin 3 heterotetramers, would also eliminate the secretion of mutant matrilin 3 into the ECM and increase the severity of chondrodysplasia in mice with the *Matn3* V194D mutation. A study testing this hypothesis would provide new insight into the disease mechanisms of MED and the role of hetero-oligomerization on the trafficking and secretion of matrilin 1/matrilin 3 proteins. We therefore crossed mice with the *Matn3* V194D (*Matn3*^m/m^) mutation with *Matn1*^−/−^ mice and generated a novel mouse line that was homozygous for the *Matn3* V194D mutation and null for matrilin 1 (*Matn1*^*−/−*^*Matn3*^m/m^). We investigated the phenotypic, morphologic, and biochemical characterization of this novel murine model to determine the interdependency of matrilin 1 and matrilin 3 oligomerization on the trafficking and secretion of these proteins and their effect on disease severity.

## MATERIALS AND METHODS

### Generation of mice

Mice with the *Matn3* V194D mutation and *Matn1*^−/−^ mice were generated as previously described (21, 27). These 2 transgenic lines were crossed to produce mice with the following genotypes: *Matn1*^+/+^*Matn3*^m/m^, *Matn1*^+/−^*Matn3*^m/m^, and *Matn1*^−/−^*Matn3*^m/m^.

### Measurement of bone lengths and monitoring of growth rates

Whole-mouse radiographs were obtained to measure the bone lengths of mice at ages 3, 6, and 9 weeks, using proprietary software (Certus Technology Associates Ltd.). Growth curves were produced by determining the body weights of the mice at 3, 6, and 9 weeks of age. One-way analysis of variance (ANOVA) was used to determine statistically significant (*P* < 0.05) differences between the different genotypes.

### Histologic and immunohistochemical analysis of growth plate cartilage

Limbs from male mice were prepared for histologic and immunohistochemical analyses, as described previously (21, 28). Sections were incubated with primary antibodies specific for matrilin 3 (R&D Systems), COMP (Genetex), type II collagen (Chemicon), matrilin 1 (ProSci), type X collagen (29), type IX collagen (30), type VI collagen (Abcam), and fibronectin (Abcam).

### Cell proliferation (bromodeoxyuridine [BrdU]) and apoptosis (TUNEL) assays on tibial growth plates

BrdU and TUNEL assays were performed as described previously (21, 28). BrdU-positive cells were counted and expressed as a percentage of the total number of cells within the proliferating zone. TUNEL-positive cells from each zone were counted and expressed as a percentage of the total number of cells within the different zones of the growth plate. Cross-sections from at least 3 different matched areas across the tibia growth plate for 3 mice of each genotype were used. Significant differences in chondrocyte proliferation and apoptosis between genotypes were tested using *t*-tests for independent samples. *P* values less than 0.05 were considered significant.

### Western blot analysis of cartilage and chondrocyte intracellular proteins

Chondrocytes were isolated from pooled rib cartilage samples obtained from 7-day-old mice, as described previously (21, 28). Chondrocyte numbers were measured using a hemocytometer, and aliquots of 1.5 × 10^5^ cells were prepared. Aliquots were spun in a microfuge for 10 minutes, supernatants were discarded, and the cell pellets were resuspended in 5× sodium dodecyl sulfate (SDS) loading buffer. Ponceau staining was used to confirm equal loading of total protein prior to blotting with antibodies specific for matrilin 3 (supplied by R&D Systems for reduced samples and a kind gift from Raimund Wagener, PhD [University of Cologne] for nonreduced samples) and matrilin 1 (a gift from Raimund Wagener). Aliquots from 2 different biologic replicates were analyzed and normalized against a β-actin control.

### Sequential cartilage extractions

Knee joints were dissected from the hind limbs of 3-week-old wild-type mice and flash frozen in liquid nitrogen. The joints were subsequently thawed and cut into ∼1-mm^3^ pieces. Tissue was extracted in 10 volumes (ml/mg wet tissue) of extraction buffer A (0.15*M* NaCl, 50 m*M* Tris [pH 7.4]) overnight at 4°C with continuous mixing. Extracts were spun in a microfuge for 10 minutes, and 100-μl aliquots of the supernatants were stored at −20°C. Insoluble material was extracted in buffer B (4*M* GuHCl, 10 m*M* EDTA, 50 m*M* Tris [pH 7.4]) as previously described, and 100-μl aliquots of the supernatants were stored at −20°C. All cartilage extractions took place in the presence of 2 m*M* phenylmethylsulfonyl fluoride and 2 m*MN*-ethylmaleimide. Supernatants from the extractions were ethanol precipitated with 96% ethanol, and the precipitated materials were spun in a microfuge for 10 minutes at 4°C. Pellets were washed with a mixture of 9 volumes of 96% ethanol and 1 volume of Tris buffered saline for 2 hours at 4°C. Precipitates were centrifuged for 10 minutes at 4°C, and the pellets were air-dried and resuspended in nonreducing SDS–polyacrylamide gel electrophoresis (PAGE) sample buffer. Twenty-microliter aliquots were analyzed by SDS-PAGE/Western blotting.

### Infrared detection of Western blots

Following Western blotting of wild-type cartilage extracts (buffer B), primary antibodies against matrilin 1 (polyclonal rabbit; a kind gift from Raimund Wagener) and matrilin 3 (polyclonal goat; R&D Systems) were applied simultaneously to the same nitrocellulose membrane. Anti-goat and anti-rabbit secondary antibodies (Li-Cor IRDye 680 and Li-Cor IRDye 800CW, respectively) were subsequently applied together to detect both matrilin 1 and matrilin 3 in the same lane of the same membrane. The Western blot was then imaged using an Odyssey Infrared Imaging System (Li-Cor).

### Cell culture primary chondrocytes

Costal chondrocytes from 5-day-old mice were extracted as described above, and the washed cell pellet was resuspended in 5 ml of cell culture medium (Dulbecco's modified Eagle's medium–Ham's F-12 supplemented with 10% fetal calf serum, 140 μ*M* ascorbate, 2 m*M*l-glutamine, 100 units/ml penicillin, and 100 units/ml streptomycin). Chondrocytes were seeded onto a 12-well plate or an 8-well Permanox Chamber Slide (Nunc) at densities of 2.5 × 10^5^ and 2 × 10^4^ cells/cm^2^, respectively, and the culture medium was replaced after 2 days. Cell lysate and culture media were collected from 12-well plate cultures after 5 days, while chamber slide cultures were fixed in 4% paraformaldehyde/phosphate buffered saline (PBS) for 10 minutes prior to immunocytochemical analysis.

### Immunocytochemical analysis of primary chondrocytes

Fixed chondrocytes were permeabilized with 0.2% Triton–PBS for 8 minutes, washed in PBS, blocked with 2% donkey serum–PBS, and incubated with primary antibodies against either matrilin 1 (Atlas Antibodies) or matrilin 3 (R&D Systems) for 1 hour. Chondrocytes were then washed with PBS, incubated with secondary antibody (Alexa Fluor 555–conjugated donkey anti-rabbit or Alexa Fluor 488–conjugated donkey anti-goat; Invitrogen), and washed in PBS before mounting in Vectashield medium with DAPI (Vector) and imaging.

## RESULTS

### Effect of matrilin 1 deletion on disease severity in mice with the *Matn3* V194D mutation

**Figure 1:**
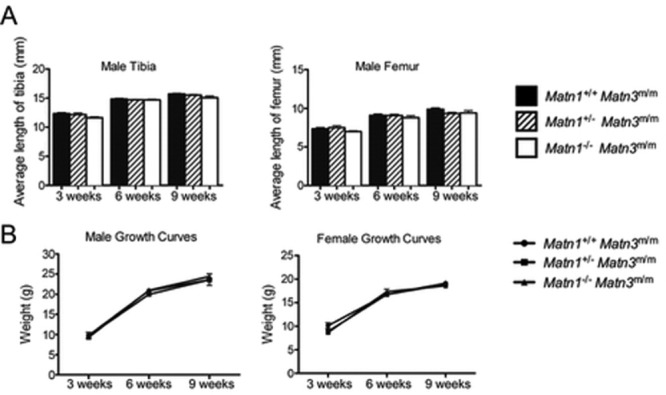
Knockout of matrilin 1 does not affect disease severity in a murine model of multiple epiphyseal dysplasia caused by a *Matn3* V194D mutation. The growth rates of *Matn1*^+/+^*Matn3*^m/m^, *Matn1*^+/−^*Matn3*^m/m^, and *Matn1*^−/−^*Matn3*^m/m^ mice were determined by analyzing bone lengths and body weights at 3, 6, and 9 weeks of age. **A,** Length of the tibia and femurs of male mice. **B,** Body weight of male and female mice. Results were analyzed by one-way analysis of variance, with no significant differences found. Values are the mean ± SEM (n > 5 mice per genotype).

To determine the phenotypic consequences of matrilin 1 knockout on the skeletal development of mice with the *Matn3* V194D mutation, radiographs were obtained at 3, 6, and 9 weeks of age and used to measure bone lengths. The tibia and femur were used to assess endochondral bone growth, while the inner canthal distance and skull length were used to monitor intramembranous ossification (Figure 1A and data not shown). The bone length measurements in mice of all 3 genotypes, *Matn1*^+/+^*Matn3*^m/m^, *Matn1*^+/−^*Matn3*^m/m^, and *Matn1*^−/−^*Matn3*^m/m^, showed no significant differences at any time point. The analyses of growth curves also revealed no phenotypic effects due to the deletion of matrilin 1 (Figure 1B).

### Effect of matrilin 1 deletion on growth plate disorganization in mice with the *Matn3* V194D mutation

**Figure 2:**
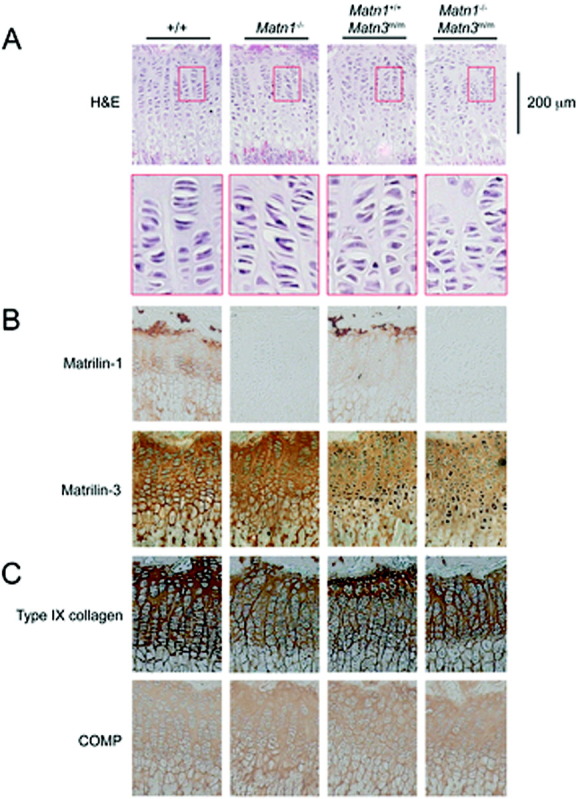
Knockout of matrilin 1 does not cause further disorganization to the cartilage growth plate or alter the localization of matrilin 3 in the extracellular matrix of mice with the *Matn3* V194D mutation. Representative images of the tibial growth plates of 3-week-old wild-type, *Matn1*^−/−^, *Matn1*^+/+^*Matn3*^m/m^, and *Matn1*^−/−^*Matn3*^m/m^ mice are shown. **A,** Top row, Organization of chondrocytes within the tibial growth plates, as visualized by hematoxylin and eosin (H&E) staining. Bottom row, Higher-magnification views of boxed areas shown in the top row. **B** and **C,** Representative photomicrographs of sections stained with antibodies specific for matrilin 1 and matrilin 3 (**B**) and for type IX collagen and cartilage oligomeric matrix protein (COMP) (**C**). Color figure can be viewed in the online issue, which is available at http://onlinelibrary.wiley.com/journal/10.1002/(ISSN)1529-0131.

Hematoxylin and eosin staining was performed to determine the effect of matrilin 1 deletion on organization of the tibial growth plates of *Matn3*^m/m^ mice (Figure 2A). The appearance and morphology of the growth plates from *Matn1*^−/−^ mice were consistent with those described in previous studies (31). Furthermore, the growth plates of 3-week-old *Matn1*^+/+^*Matn3*^m/m^ and *Matn1*^−/−^*Matn3*^m/m^ mice showed disruption to the morphology and organization of chondrocytes, which was comparable with our previous findings in *Matn3*^m/m^ mice (21). For example, chondrocytes in the proliferative zone in both genotypes appeared misshapen, and their arrangement into chondrons was disturbed when compared with wild-type and *Matn1*^−/−^ control mice (Figure 2A).

### Secretion of mutant matrilin 3 into the ECM in the absence of matrilin 1

Given the fact that matrilin 1 and matrilin 3 monomers form hetero-oligomers, we used immunohistochemistry to analyze the tibial growth plates of 3-week-old mice to determine whether the deletion of matrilin 1 might abrogate the secretion of mutant matrilin 3. In wild-type and *Matn1*^−/−^ mice, normal matrilin 3 was secreted into the ECM, as expected. In contrast, intracellular retention of mutant matrilin 3 was observed in mice with the *Matn3* V194D mutation that were either null or wild-type for matrilin 1 (i.e., *Matn1*^+/+^*Matn3*^m/m^ and *Matn1*^−/−^*Matn3*^m/m^ mice, respectively) (Figure 2B). However, it was also obvious that a proportion of mutant matrilin 3 was still secreted into the ECM independent of matrilin 1. In wild-type and *Matn1*^−/−^ mice, staining for matrilin 3 appeared more pronounced in the pericellular matrix surrounding the chondrons. In contrast, staining for mutant matrilin 3 was more evenly distributed in the interterritorial regions between chondrocyte columns of growth plate cartilage in *Matn1*^+/+^*Matn3*^m/m^ and *Matn1*^−/−^*Matn3*^m/m^ mice.

### Effect of matrilin 1 deletion on the localization of other ECM proteins in the growth plates of mice with the *Matn3* V194D mutation

Given the predicted role of the matrilin proteins as adaptor molecules, we used immunohistochemistry to probe the localization of key ECM molecules in *Matn1*^−/−^*Matn3*^m/m^ mice. At 3 weeks of age, the spatial localization of collagen types II, VI, IX, and X, COMP, and fibronectin were comparable between *Matn1*^−/−^*Matn3*^m/m^ and *Matn1*^+/+^*Matn3*^m/m^ mice (Figure 2C and data not shown). Furthermore, there were no apparent differences in the localization of these key proteins due to the deletion of *Matn1* alone, with the exception of type IX collagen, the staining for which was consistently higher at the boundary between the resting zone and secondary center of ossification in wild-type and *Matn1*^+/+^*Matn3*^m/m^ mice. This observation mirrors the increased staining for matrilin 1 at the same location (Figure 2B) and suggests that these proteins interact in vivo.

### Effect of matrilin 1 deletion on the reduced rate of chondrocyte proliferation in mice with the *Matn3* V194D mutation

**Figure 3:**
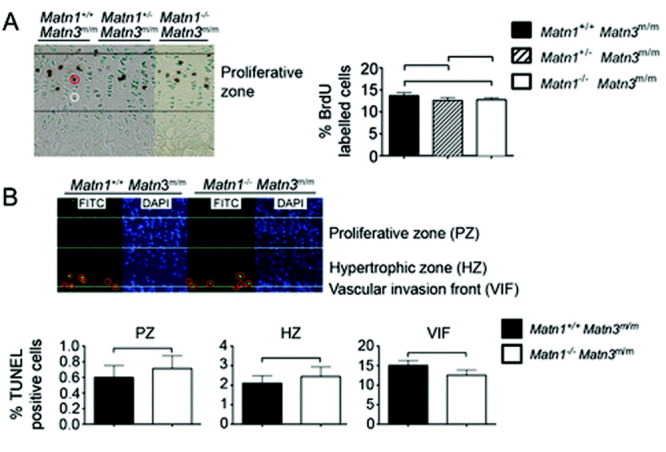
Knockout of matrilin 1 does not affect the rate of chondrocyte proliferation or the levels and spatial localization of chondrocyte apoptosis in the growth plates of mice with the *Matn3* V194D mutation. **A,** Chondrocyte proliferation was visualized in the tibial growth plates of 3-week-old mice, using an antibody specific for bromodeoxyuridine (BrdU). Representative images of tibial growth plates show a BrdU-labeled proliferating chondrocyte (red circle) and a nonproliferating chondrocyte stained with methyl green (white circle). The relative percentage of proliferating cells within the tibial growth plates of mice was determined. **B,** The DNA fragmentation stage of apoptosis was measured in the tibial growth plates of 3-week-old mice by TUNEL analysis. Representative images of growth plate sections show TUNEL-positive chondrocytes (red circles) and DAPI-stained chondrocyte nuclei. The percentage of TUNEL-positive cells within the growth plate zones of *Matn1*^+/+^*Matn3*^m/m^ and *Matn1*^−/−^*Matn3*^m/m^ mouse tibia was determined. Bars show the mean ± SEM. FITC = fluorescein isothiocyanate. Color figure can be viewed in the online issue, which is available at http://onlinelibrary.wiley.com/journal/10.1002/(ISSN)1529-0131.

We previously showed that reduced chondrocyte proliferation in the growth plate was a key disease mechanism in a murine model of MED caused by a *Matn3* V194D mutation (21). We therefore performed BrdU labeling to determine whether the deletion of matrilin 1 would further reduce the rate of chondrocyte proliferation (Figure 3A). One-way ANOVA revealed no statistically significant differences between the proportions of proliferating chondrocytes in mice of all 3 genotypes.

### Influence of matrilin 1 deletion on spatially dysregulated chondrocyte apoptosis in mice with the *Matn3* V194D mutation

We previously showed that dysregulated chondrocyte apoptosis in the growth plate is another key disease mechanism in mice with the *Matn3* V194D mutation (21). We therefore calculated the relative rates of chondrocyte apoptosis within the tibial growth plates of 3-week-old mice to determine whether the deletion of matrilin 1 influences chondrocyte apoptosis (Figure 3B). Based on these data, it was evident that the relative levels of apoptosis within the different growth plate zones were not significantly different between *Matn1*^+/+^*Matn3*^m/m^ and *Matn1*^−/−^*Matn3*^m/m^ mice, when using *t*-tests for independent samples.

### Molecular characterization of retained mutant matrilin 3

**Figure 4:**
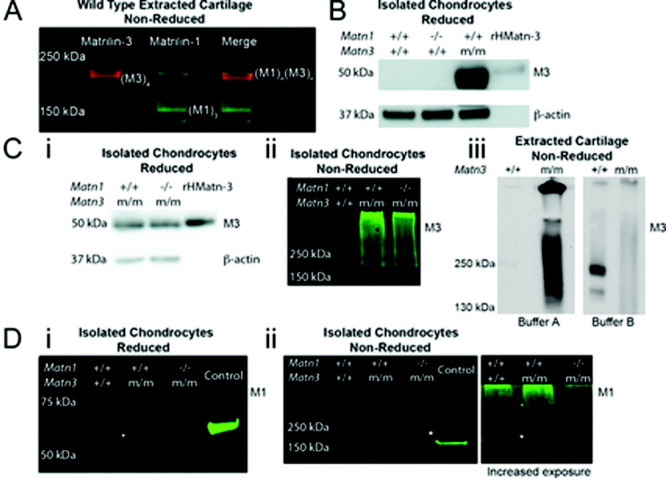
Matrilin 3 forms heterotetramers with matrilin 1, but the level of retained mutant matrilin 3 is not increased by the loss of matrilin 1 in chondrocytes from mice with the *Matn3* V194D mutation. **A,** Total cartilage proteins from *Matn1*^+/+^*Matn3*^+/+^ mice were resolved by nonreducing sodium dodecyl sulfate–polyacrylamide gel electrophoresis (SDS-PAGE)/Western blotting and visualized by direct infrared fluorescence detection. Merging of matrilin 3 (red) and matrilin 1 (green) staining demonstrated the presence of matrilin 1/matrilin 3 heterotetramers (M1)_n_(M3)_n_ (orange). **B,** Intracellular matrilin 3 was detected only in *Matn1*^+/+^*Matn3*^m/m^ mouse chondrocytes and not in *Matn1*^+/+^*Matn3*^+/+^ or *Matn1*^−/−^*Matn3*^+/+^ controls. **C, i,** There were no obvious differences in the levels of retained mutant matrilin 3 in cells from *Matn1*^+/+^*Matn3*^m/m^ mice and those from *Matn1*^−/−^*Matn3*^m/m^ mice. **ii,** Retained intracellular mutant matrilin 3 was analyzed by nonreducing SDS-PAGE of isolated chondrocyte protein lysate. **iii,** SDS-PAGE analysis of extracted cartilage proteins confirmed that mutant matrilin 3 was extracted as a Tris–NaCl soluble aggregate (m/m in buffer A). In contrast, wild-type matrilin 3 was extracted as discrete tetramers and trimers using GuHCl (+/+ in buffer B). **D,** A proportion of wild-type matrilin 1 was co-retained with mutant matrilin 3 in *Matn1*^+/+^*Matn3*^m/m^ mouse chondrocytes under reducing conditions (**i**), which was consistent in size with the (M1)_n_(M3)_n_ hetero-oligomers (*) when analyzed under nonreducing conditions and the image exposure was increased (**ii**). rHMatn-3 = recombinant human *Matn3*.

It has previously been demonstrated that (matrilin 1)_2_(matrilin 3)_2_ heterotetramers can be isolated from human and bovine cartilage (23, 24, 26), but the existence of this molecular form in mouse cartilage has not been convincingly demonstrated to date (32). We therefore performed Western blot analysis of GuHCl-extracted mouse cartilage proteins, using direct infrared fluorescence detection with a Li-Cor Odyssey Imaging System, to confirm the existence of matrilin 1/matrilin 3 heterotetramers in mouse cartilage (Figure 4A). However, it was clear from this experiment that these heterotetramers were less abundant than the respective homotrimers and homotetramers of matrilin 1 and matrilin 3.

We hypothesized that the deletion of matrilin 1 might increase the relative amount of mutant matrilin 3 retained within chondrocytes by preventing the formation and secretion of these matrilin 1 (wild-type)/matrilin 3 (mutant) hetero-oligomers. We tested this hypothesis by isolating chondrocytes from the pooled rib cartilage specimens of 7-day-old littermates (∼5–7 pups per litter) and analyzing the intracellular proteins by SDS-PAGE and Western blotting. The complete absence of any detectable matrilin 3 in the wild-type cell extracts demonstrated that the cellular protein preparations were not contaminated with any secreted matrilin 3 protein derived from the ECM (Figure 4B). Subsequent experiments reproducibly showed that the relative amounts of intracellular mutant matrilin 3 protein did not differ between *Matn1*^+/+^*Matn3*^m/m^ mice and *Matn1*^−/−^*Matn3*^m/m^ mice (Figure 4C, part i).

In order to determine the oligomeric state of the retained mutant matrilin 3, we also analyzed nonreduced protein samples, using SDS-PAGE. This analysis indicated that although some of the retained mutant matrilin 3 was present as distinct oligomers, the vast majority of mutant protein existed as high molecular weight disulfide-bonded aggregates (Figure 4C, part ii) independent of the presence or absence of matrilin 1. This aggregated form of mutant matrilin 3 was confirmed by examining sequential protein extracts from total mice cartilage (Figure 4C, part iii), which showed that mutant matrilin 3 was readily extracted by Tris–NaCl (buffer A)**,** while GuHCl (buffer B) was required to extract wild-type matrilin 3. In addition, wild-type matrilin 3 appeared to exist in 2 oligomeric forms that corresponded in size to trimers and tetramers; however, tetramers were clearly the more prevalent form.

A proportion of matrilin 1 was co-retained with mutant matrilin 3 in *Matn1*^+/+^*Matn3*^m/m^ mouse chondrocytes (Figure 4D, part i), which appeared consistent in size with the putative (matrilin 1)_2_(matrilin 3)_2_ heterotetramer extracted from wild-type cartilage when analyzed under nonreducing conditions and the image exposure was increased (Figure 4D, part ii). The observation of apparently high molecular weight matrilin 1–containing aggregates most likely arose from the increased exposure of the image, and such aggregates were not observed upon reduction.

### Molecular characterization of secreted mutant matrilin 3

**Figure 5:**
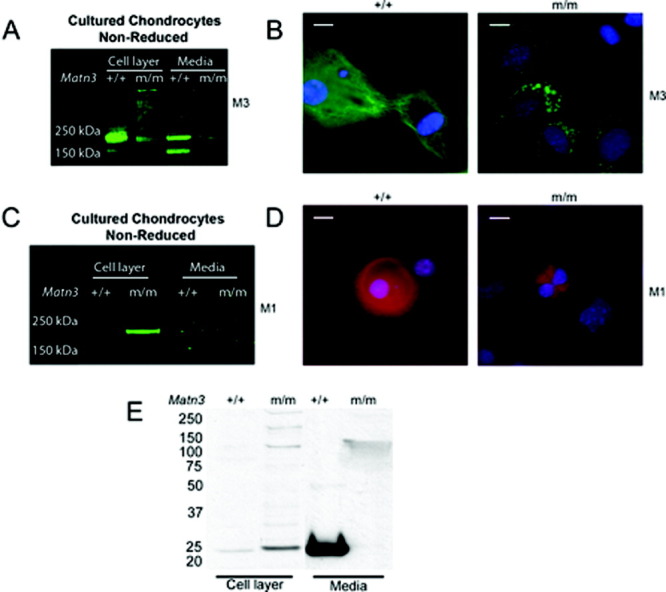
Mutant matrilin 3 secreted by cultured chondrocytes exists as a tetramer, and the aggregation of retained mutant matrilin 3 is triggered by misfolded A domains. The cell layer and culture media from primary chondrocytes were analyzed by Western blotting and fluorescence microscopy. **A,** Nonreducing sodium dodecyl sulfate–polyacrylamide gel electrophoresis (SDS-PAGE)/Western blotting showed that mutant matrilin 3 could be secreted as a putative tetramer (m/m in media) and also confirmed that mutant matrilin 3 was retained as a high molecular weight aggregate (m/m in cell layer). **B,** Retained intracellular mutant matrilin 3 (m/m) showed a punctate appearance when analyzed by fluorescence microscopy, in contrast to the filamentous network–like structure of matrilin 3 secreted by wild-type chondrocytes (+/+). **C** and **D,** Nonreducing SDS-PAGE/Western blotting and fluorescence microscopy, respectively, confirmed the co-retention of wild-type matrilin 1 in V194D mouse chondrocytes (m/m). **E,** A domain DNA of wild-type (+/+) and matrilin 3 V194D (m/m) was cloned and expressed in HEK 293 cells. Cell-layer and media fractions were separated by SDS-PAGE under nonreducing conditions, and the recombinant A domains were detected by Western blotting using an anti–FLAG antibody. The V194D mutant A domains were retained within the cell layer and formed a regular pattern of disulfide-bonded aggregates (m/m). Bars in **B** and **D** = 10 μm. M1 = matrilin 1; M3 = matrilin 3. Color figure can be viewed in the online issue, which is available at http://onlinelibrary.wiley.com/journal/10.1002/(ISSN)1529-0131.

To determine the molecular organization of secreted mutant matrilin 3, we analyzed the cell/cell-layer and media proteins of cultured mouse chondrocytes (Figure 5A). Western blot analysis of wild-type samples showed the presence of trimers and tetramers, which was consistent with the molecular forms observed following GuHCl extraction of cartilage; in both cases, tetramers were the predominant form. Fluorescence microscopy confirmed that wild-type matrilin 3 was present in pericellular networks, and that there was no intracellular retention (Figure 5B). In contrast, mutant matrilin 3 existed predominantly in a tetrameric form in both the cell/cell-layer and media of cultured *Matn1*^+/+^*Matn3*^m/m^ chondrocytes (Figure 5A), while the presence in the cell/cell-layer fraction of larger aggregates was comparable with that observed in isolated chondrocytes. Furthermore, intracellular retention of mutant matrilin 3 was verified by fluorescence microscopy, which demonstrated a punctate appearance and also confirmed the absence of matrilin 3 from the pericellular networks (Figure 5B).

Matrilin 1 was present in a tetrameric form in both the cell/cell-layer and media samples of cultured *Matn1*^+/+^*Matn3*^+/+^ chondrocytes (Figure 5C), and the pericellular localization of matrilin 1 was confirmed by fluorescence microscopy (Figure 5D). Finally, these studies confirmed the retention of considerable amounts of matrilin 1 in the cell/cell-layer fraction of cultured *Matn1*^+/+^*Matn3*^m/m^ chondrocytes (Figure 5C), which also appeared to have an intracellular punctate appearance (Figure 5D).

### Possible mechanism of aggregation of mutant matrilin 3 oligomers

We previously demonstrated the intracellular retention of recombinant mutant V194D matrilin 3 A domains following reducing SDS-PAGE (15). Here, we showed that the retained V194D mutant A domains formed a regular pattern of disulfide-bonded aggregates (Figure 5E), which could be resolved to a single band following reduction. This observation was consistent with the disulfide-bonded aggregates of full-length mutant matrilin isolated from *Matn1*^+/+^*Matn3*^m/m^ chondrocytes and cartilage (Figure 4C) and points to the misfolded mutant A domain as the nucleation site for the formation of the non-native disulfide-bonded full-length mutant matrilin 3 aggregates.

## DISCUSSION

In this study, we explored the effect of *Matn1* deletion on the phenotype of mice that were homozygous for a MED-causing matrilin 3 mutation (*Matn3* V194D) (21). We hypothesized that the relatively low levels of mutant matrilin 3 in the cartilage ECM of mice with the *Matn3* V194D mutation might be secreted through the formation of (matrilin 1^+/+^)_n_(matrilin 3^m/m^)_n_ hetero-oligomers. The formation of a hetero-oligomeric form of matrilin 1/matrilin 3 in vivo has previously been demonstrated for human and bovine cartilage (23–26) and is now confirmed for mice in this study, but its physiologic relevance and the interdependency of matrilin 1 and matrilin 3 monomers in oligomerization, protein trafficking, and secretion remain unknown.

We evaluated the phenotype of *Matn1*^−/−^*Matn3*^m/m^ mice and demonstrated that the deletion of *Matn1* had no effect on the relative growth rate and long bone lengths of mice with the *Matn3* V194D mutation. These observations are in accordance with findings for *Matn1*-knockout mice, which showed no overt skeletal phenotype (31). Our analyses also confirmed that there were no further differences in the relative levels of chondrocyte proliferation and apoptosis within the growth plates of mice with the *Matn3* V194D mutation due to the deletion of *Matn1*. We previously proposed that these detrimental changes in the chondrocyte phenotype are key disease mechanisms in MED caused by the *Matn3* V194D mutation (21, 22) and also in pseudoachondroplasia–MED caused by *Comp* p.T585M (28) and *Comp* p.D469del (33) mutations. Overall, these data suggest that changes in the levels of matrilin 1 expression would not modify disease severity in patients with MED and therefore do not account for the interfamilial variability seen in some families with MED and *MATN3* mutations (34).

The analysis of growth plate morphology and the spatial distribution of key structural proteins such as type IX collagen and COMP showed some variation between mice of different genotypes, possibly because matrilin 1/matrilin 3, COMP, and type IX collagen have been shown to interact in vitro (7, 13). For example, there was reduced staining for type IX collagen at the boundary between the resting zone and the secondary center of ossification in the growth plates of mice that lacked matrilin 1. This reduced staining for type IX collagen was inversely proportional to the higher levels of staining for matrilin 1 in the same region in wild-type and *Matn1*^+/+^*Matn3*^m/m^ mice, suggesting that these 2 molecules interact in vivo, and that matrilin 1 is important for anchoring type IX collagen into the ECM. This observation confirms recent in vitro binding studies showing interactions between type IX collagen and the A domains of matrilin 1 (13). Type IX collagen–deficient mice also showed altered integration of matrilin 3 into the ECM (10), confirming that these proteins interact in vivo*.*

Interestingly, immunohistochemical analysis revealed that although staining for wild-type matrilin 3 was localized primarily to the pericellular matrix surrounding chondrocytes, mutant matrilin 3 staining was spread evenly throughout the territorial matrix between chondrocyte columns, irrespective of the matrilin 1 genotype. Similar observations were made in the original characterization of mice with the *Matn3* V194D mutation and may suggest that the secreted mutant matrilin 3 is less able to incorporate correctly into the pericellular matrix. Sequential extractions of mutant mouse cartilage showed increased extractability of mutant matrilin 3, which was present predominantly as disulfide-bonded high molecular weight aggregates; however, this approach is limited, because it is likely that extracted intracellular mutant proteins will mask ECM-derived proteins. In contrast, wild-type matrilin 3 was extracted only with GuHCl, suggesting that it is tightly integrated into the ECM. Indeed, fluorescence microscopy of cultured wild-type mouse chondrocytes showed that matrilin 3 was present in filamentous networks surrounding the cells.

We also used this novel murine model to probe the nature of matrilin 1/matrilin 3 hetero-oligomers in vivo. Immunohistochemical analysis and Western blotting of intracellular proteins were used to test the hypothesis that the ablation of matrilin 1 would alter the secretion of matrilin 3 V194D and result in increased levels of retained mutant matrilin 3. However, immunohistochemistry showed that the deletion of matrilin 1 did not abrogate the secretion of mutant matrilin 3, because ECM staining for mutant matrilin 3 was similar in *Matn1*^+/+^*Matn3*^m/m^ and *Matn1*^−/−^*Matn3*^m/m^ mice. Western blotting of extracted intracellular proteins from *Matn1*^+/+^*Matn3*^m/m^ and *Matn1*^−/−^*Matn3*^m/m^ chondrocytes confirmed that the intracellular levels of retained mutant matrilin 3 protein were not altered. Furthermore, the analysis of cultured *Matn1*^+/+^*Matn3*^m/m^ chondrocytes confirmed that a small proportion of mutant matrilin 3 was secreted as a putative tetramer, although the majority still existed in punctate intracellular inclusions.

Intracellular matrilin 1 was also detected at low levels by Western blotting of total chondrocyte protein following reducing SDS-PAGE. The results of SDS-PAGE run under nonreducing conditions suggested that these oligomers might represent (matrilin 1)_2_(matrilin 3)_2_ heterotetramers. This observation suggests that matrilin 1 monomers are being retained due to their association with mutant matrilin 3 monomers in heterotetramers. Although we could not demonstrate any phenotypic differences between *Matn1*^+/+^*Matn3*^m/m^ and *Matn1*^−/−^*Matn3*^m/m^ mice, we cannot exclude the possibility that the retention of these hetero-oligomers, along with the more prevalent matrilin 3 homo-oligomers, contributes to the etiology of MED. The apparent differences in the levels of retained matrilin 1 between isolated and cultured chondrocytes are attributable to the use of collagenase for the isolation of ECM-free chondrocytes. In contrast, both intracellular and cell-layer proteins (pericellular) are collected from cultured chondrocytes by cell scraping (i.e., “cell-layer” samples). Overall, these findings suggest that the majority of intracellular and extracellular mutant matrilin 3 appears to be in the form of (matrilin 3)_4_, and that (matrilin 1)_wild-type_(matrilin 3)_mutant_ hetero-oligomers represent only a small fraction, which is consistent with the relative proportion of wild-type heterotetramers compared with the respective homotrimers and homotetramers of matrilin 1 and matrilin 3.

Finally, using nonreducing SDS-PAGE and Western blotting, we showed that the retained mutant matrilin 3 forms disulfide-bonded aggregates in chondrocytes, and that the mutant A domain alone (V194D) also formed similar disulfide-bonded aggregates in vitro. We previously demonstrated that various missense mutations in the β-strands of the A domain delayed folding and prevented correct intramolecular disulfide bond formation (15). In this study, we extended these findings and demonstrated that the mutant A domains form aggregates through non-native intermolecular disulfide bonds. It is likely that the increased expression of several members of the protein disulfide isomerase–associated protein family (i.e., *Pdia4*, *Pdia5*, and *Pdia6*) in chondrocytes from mice with the *Matn3* V194D mutation (22) may be the direct result of incorrect disulfide bond formation between mutant matrilin 3 monomers nucleated at the A domain.

In conclusion, we explored the effect of matrilin 1 deletion in mice homozygous for a MED-causing matrilin 3 mutation. We observed no alteration to the MED-like phenotype of mutant mice and no relative increase in the quantity of intracellular mutant matrilin 3, suggesting that mutant matrilin 3 exists primarily as a homo-oligomer. The observation that chondrocytes can potentially fold and secrete a proportion of mutant matrilin 3 oligomers supports the idea that treatment with small molecular chaperones might aid in the secretion of mutant matrilin 3 and pave the way for the development of suitable therapies (22).

## References

[1] Superti-Furga A, Unger S (2007). Nosology and classification of genetic skeletal disorders: 2006 revision. Am J Med Genet A.

[2] Kornak U, Mundlos S (2003). Genetic disorders of the skeleton: a developmental approach. Am J Hum Genet.

[3] Unger S, Bonafe L, Superti-Furga A (2008). Multiple epiphyseal dysplasia: clinical and radiographic features, differential diagnosis and molecular basis. Best Pract Res Clin Rheumatol.

[4] Briggs MD, Chapman KL (2002). Pseudoachondroplasia and multiple epiphyseal dysplasia: mutation review, molecular interactions, and genotype to phenotype correlations. Hum Mutat.

[5] Jackson GC, Mittaz-Crettol L, Taylor JA, Mortier GR, Spranger J, Zabel B (2012). Pseudoachondroplasia and multiple epiphyseal dysplasia: a 7-year comprehensive analysis of the known disease genes identify novel and recurrent mutations and provides an accurate assessment of their relative contribution. Hum Mutat.

[6] Wagener R, Ehlen HW, Ko YP, Kobbe B, Mann HH, Sengle G (2005). The matrilins: adaptor proteins in the extracellular matrix. FEBS Lett.

[7] Fresquet M, Jowitt TA, Ylostalo J, Coffey P, Meadows RS, Ala-Kokko L (2007). Structural and functional characterization of recombinant matrilin 3 A domain and implications for human genetic bone diseases. J Biol Chem.

[8] Blumbach K, Niehoff A, Paulsson M, Zaucke F (2008). Ablation of collagen IX and COMP disrupts epiphyseal cartilage architecture. Matrix Biol.

[9] Blumbach K, Bastiaansen-Jenniskens YM, DeGroot J, Paulsson M, van Osch GJ, Zaucke F (2009). Combined role of type IX collagen and cartilage oligomeric matrix protein in cartilage matrix assembly: cartilage oligomeric matrix protein counteracts type IX collagen–induced limitation of cartilage collagen fibril growth in mouse chondrocyte cultures. Arthritis Rheum.

[10] Budde B, Blumbach K, Ylostalo J, Zaucke F, Ehlen HW, Wagener R (2005). Altered integration of matrilin 3 into cartilage extracellular matrix in the absence of collagen IX. Mol Cell Biol.

[11] Zaucke F, Grassel S (2009). Genetic mouse models for the functional analysis of the perifibrillar components collagen IX, COMP and matrilin 3: implications for growth cartilage differentiation and endochondral ossification. Histol Histopathol.

[12] Holden P, Meadows RS, Chapman KL, Grant ME, Kadler KE, Briggs MD (2001). Cartilage oligomeric matrix protein interacts with type IX collagen, and disruptions to these interactions identify a pathogenetic mechanism in a bone dysplasia family. J Biol Chem.

[13] Fresquet M, Jowitt TA, Stephen LA, Ylostalo J, Briggs MD (2010). Structural and functional investigations of Matrilin-1 A-domains reveal insights into their role in cartilage ECM assembly. J Biol Chem.

[14] Deak F, Wagener R, Kiss I, Paulsson M (1999). The matrilins: a novel family of oligomeric extracellular matrix proteins. Matrix Biol.

[15] Cotterill SL, Jackson GC, Leighton MP, Wagener R, Makitie O, Cole WG (2005). Multiple epiphyseal dysplasia mutations in MATN3 cause misfolding of the A-domain and prevent secretion of mutant matrilin 3. Hum Mutat.

[16] Chapman KL, Mortier GR, Chapman K, Loughlin J, Grant ME, Briggs MD (2001). Mutations in the region encoding the von Willebrand factor A domain of matrilin 3 are associated with multiple epiphyseal dysplasia. Nat Genet.

[17] Jackson GC, Barker FS, Jakkula E, Czarny-Ratajczak M, Makitie O, Cole WG (2004). Missense mutations in the beta strands of the single A-domain of matrilin 3 result in multiple epiphyseal dysplasia. J Med Genet.

[18] Mabuchi A, Haga N, Maeda K, Nakashima E, Manabe N, Hiraoka H (2004). Novel and recurrent mutations clustered in the von Willebrand factor A domain of MATN3 in multiple epiphyseal dysplasia. Hum Mutat.

[19] Fresquet M, Jackson GC, Loughlin J, Briggs MD (2008). Novel mutations in exon 2 of MATN3 affect residues within the α-helices of the A-domain and can result in the intracellular retention of mutant matrilin 3. Hum Mutat.

[20] Otten C, Wagener R, Paulsson M, Zaucke F (2005). Matrilin-3 mutations that cause chondrodysplasias interfere with protein trafficking while a mutation associated with hand osteoarthritis does not. J Med Genet.

[21] Leighton MP, Nundlall S, Starborg T, Meadows RS, Suleman F, Knowles L (2007). Decreased chondrocyte proliferation and dysregulated apoptosis in the cartilage growth plate are key features of a murine model of epiphyseal dysplasia caused by a matn3 mutation. Hum Mol Genet.

[22] Nundlall S, Rajpar MH, Bell PA, Clowes C, Zeeff LA, Gardner B (2010). An unfolded protein response is the initial cellular response to the expression of mutant matrilin-3 in a mouse model of multiple epiphyseal dysplasia. Cell Stress Chaperones.

[23] Kleemann-Fischer D, Kleemann GR, Engel D, Yates JR, Wu JJ, Eyre DR (2001). Molecular properties of matrilin-3 isolated from human growth cartilage. Arch Biochem Biophys.

[24] Wu JJ, Eyre DR (1998). Matrilin-3 forms disulfide-linked oligomers with matrilin 1 in bovine epiphyseal cartilage. J Biol Chem.

[25] Frank S, Schulthess T, Landwehr R, Lustig A, Mini T, Jeno P (2002). Characterization of the matrilin coiled-coil domains reveals seven novel isoforms. J Biol Chem.

[26] Klatt AR, Nitsche DP, Kobbe B, Morgelin M, Paulsson M, Wagener R (2000). Molecular structure and tissue distribution of matrilin 3, a filament-forming extracellular matrix protein expressed during skeletal development. J Biol Chem.

[27] Hyde G, Dover S, Aszodi A, Wallis GA, Boot-Handford RP (2007). Lineage tracing using matrilin-1 gene expression reveals that articular chondrocytes exist as the joint interzone forms. Dev Biol.

[28] Pirog-Garcia KA, Meadows RS, Knowles L, Heinegard D, Thornton DJ, Kadler KE (2007). Reduced cell proliferation and increased apoptosis are significant pathological mechanisms in a murine model of mild pseudoachondroplasia resulting from a mutation in the C-terminal domain of COMP. Hum Mol Genet.

[29] Rajpar MH, McDermott B, Kung L, Eardley R, Knowles L, Heeran M (2009). Targeted induction of endoplasmic reticulum stress induces cartilage pathology. PLoS Genet.

[30] Douglas SP, Jenkins JM, Kadler KE (1998). Collagen IX: evidence for a structural association between NC4 domains in cartilage and a novel cleavage site in the α 1(IX) chain. Matrix Biol.

[31] Aszodi A, Bateman JF, Hirsch E, Baranyi M, Hunziker EB, Hauser N (1999). Normal skeletal development of mice lacking matrilin-1: redundant function of matrilins in cartilage?. Mol Cell Biol.

[32] Nicolae C, Ko YP, Miosge N, Niehoff A, Studer D, Enggist L (2007). Abnormal collagen fibrils in cartilage of matrilin-1/matrilin-3-deficient mice. J Biol Chem.

[33] Suleman F, Gualeni B, Gregson HG, Leighton MP, Pirog KA, Edwards S (2012). A novel form of chondrocyte stress is triggered by a COMP mutation causing pseudoachondroplasia. Hum Mutat.

[34] Makitie O, Mortier GR, Czarny-Ratajczak M, Wright MJ, Suri M, Rogala P (2004). Clinical and radiographic findings in multiple epiphyseal dysplasia caused by MATN3 mutations: description of 12 patients. Am J Med Genet.

